# Utilizing biomaterial surface properties to improve orthopedic hip implant safety and function in a Safe-by-Design approach

**DOI:** 10.3389/fbioe.2025.1504883

**Published:** 2025-02-21

**Authors:** Anniek M. C. Gielen, Niels M. Leijten, Payal P. S. Balraadjsing, Hedwig M. Braakhuis, Hannah Abee, Jacobus J. Arts, Annemarie P. van Wezel, Agnes G. Oomen, Nick R. M. Beijer

**Affiliations:** ^1^ National institute of Public Health and the Environment, Bilthoven, Netherlands; ^2^ Institute for Biodiversity and Ecosystem Dynamics, University of Amsterdam, Amsterdam, Netherlands; ^3^ Department of Medical Microbiology and Infection Prevention, Amsterdam UMC, Amsterdam Institute for Immunology and Infection Diseases, University of Amsterdam, Amsterdam, Netherlands; ^4^ Department of Orthopaedic Surgery, Maastricht UMC+, Maastricht University Medical Centre, Maastricht, Netherlands; ^5^ Department of Biomedical Engineering, Eindhoven University of Technology, Eindhoven, Netherlands

**Keywords:** osseointegration, immune response, bacterial adhesion, surface properties, Safe-by-Design

## Abstract

Orthopedic hip implant failure due to adverse events, such as infection, are still a major problem leading to high morbidity and mortality. Over the years, various innovative biomaterials have been investigated to improve safety and functionality of implants. Although novel biomaterials show initial promising results, many fail at the (later) stages of safety testing. We performed a literature review serving as a first step in a Safe-by-Design (SbD) approach. SbD is a strategy which includes safety considerations at early development stages and that streamlines the pre-clinical safety assessment of innovative medical implants. In a SbD approach, the standard safety assessment of medical implants (e.g., ISO10993) is complemented with insights on cell-biomaterial interactions allowing for a better *in vivo* response prediction. As a first step, these insights are based on existing information from literature. Therefore, in this review, correlations between implant biomaterial surface properties and key biological processes, relevant for the success and safety of titanium hip implants, are investigated. In particular, the influence of biomaterial roughness, wettability and pore size on key biological processes for a hip implant (osseointegration, bacterial adhesion and the immune response) are examined. Although it was found that no ideal combination of properties exist to satisfy the key biological processes simultaneously, the gathered insights provide directions for the development of safe and functional biomaterials. Altogether, an assessment of the different aspects of safety at early development stages within an SbD approach can improve biomaterial functionality and thus safety.

## 1 Introduction

Orthopedic hip implants are widely used medical devices that can contribute immensely to the quality of life of patients. In the Netherlands, yearly, over 30 thousand hip replacements were performed (LROI DAR, 2020), for a population of 16,8 to 17,2 million between 2014 and 2019 respectively (Netherlands, 2024). The annual need for implants is expected to grow due to an aging population. Besides the positive contributions, there is a potential risk of adverse outcomes such as implant infection or implant loosening. Adverse outcomes after implantation are common causes of revision. Based on Dutch data between 2014 and 2019, for 1 out of 8 hip replacements, revisions were performed, with 22.8% due to loosening of the acetabulum component (surface in the pelvis), 19.1% attributed to infection and 18.9% to loosening of the femur component (LROI DAR, 2020). These adverse outcomes pose a health risk to the patient and increase the economic burden to the healthcare system (OECD, 2021). Adverse outcomes are influenced by patient characteristics, surgeon expertise as well as implant design. While retrospective studies on implant failure often focus on factors like gender, fracture type and femur size, they rarely consider implant material design (Roerink et al., 2024).

It is important that biological processes that influence adverse outcomes are considered during the safety assessment of orthopedic hip implants. Knowledge on key biological processes can feed into a Safe-by-Design (SbD) framework and be implemented into the design phase (Schmutz et al., 2020). For example, implant infection occurs when microorganisms adhere and colonize the implant surface and surrounding tissue, leading to tissue damage and loss of implant function (Rosman et al., 2021). Loosening of the femur component could be due to lack of osseointegration or due to a strong inflammatory response, compromising surrounding tissue (Nobles et al., 2021). Implementing key biological processes such as bacterial adhesion and osseointegration early into the design phase of novel implants allows innovators to incorporate safety at an early stage of development. SbD can help to streamline innovation and ultimately increase patient safety.

In this review, we propose a Safe-by-Design approach for medical implants, focusing on innovative antimicrobial orthopedic hip implants, with titanium as primary focus biomaterial including modifications by coating. We structure existing information that can be used as a first step in SbD. To that end, literature reviews (listed in Supplementary Table S1) focusing on one of three well-studied biomaterial surface properties (roughness, wettability and pore size),are assessed to determine their relationship to three key biological processes (osseointegration, bacterial adhesion and immune response). This approach will allow to identify directions for implant biomaterial development at early innovation stages, that can be further detailed by *in vitro* and ultimately *in vivo* testing related to these key biological processes.

Literature search strings have been developed tailored to various combinations of biomaterial properties and biological processes. PubMed and Embase served as the primary databases for this effort. Snowball citations were employed to identify additional relevant literature. Exclusion criteria were established to filter out studies involving mandibular implantation, skull implantation and induced animal models.

## 2 Safe-by-Design for functional and safe implants

Safe and Sustainable by Design (SSbD) is currently actively promoted by the European Commission as part of the European Green Deal and the Chemicals Strategy for Sustainability to strive for a prosperous society without harm to humans or the environment caused by hazardous materials or chemicals (European et al., 2022a; European et al., 2022b). The SSbD concept refers to anticipating risks and uncertainties concerning human and environmental safety early on in the innovation and development process. It addresses the safety and sustainability of the material/product as well as the associated processes throughout the whole cycle of innovation. Safety and sustainability is integrated in product development in an iterative manner to include information on safety, functionality and other aspects such as costs or sustainability early on. It thereby prevents as much as possible, failure later on in the development process (van de Poel and Robaey, 2017; Tavernaro et al., 2021; van Gelder et al., 2021). This paper will primarily focus on safety considerations, within the framework of Safe-by-Design (SbD). While sustainability remains an important topic, our initial emphasis will be directed towards ensuring patient safety by minimizing adverse outcomes. SbD was first introduced in the nanotechnology field to address potential risks induced by novel nanomaterials (Dekkers et al., 2020), and has been introduced in other sectors (e.g., chemical industry) (van Dijk et al., 2022). This paper applies the SbD principles to medical implants.

**FIGURE 1:**
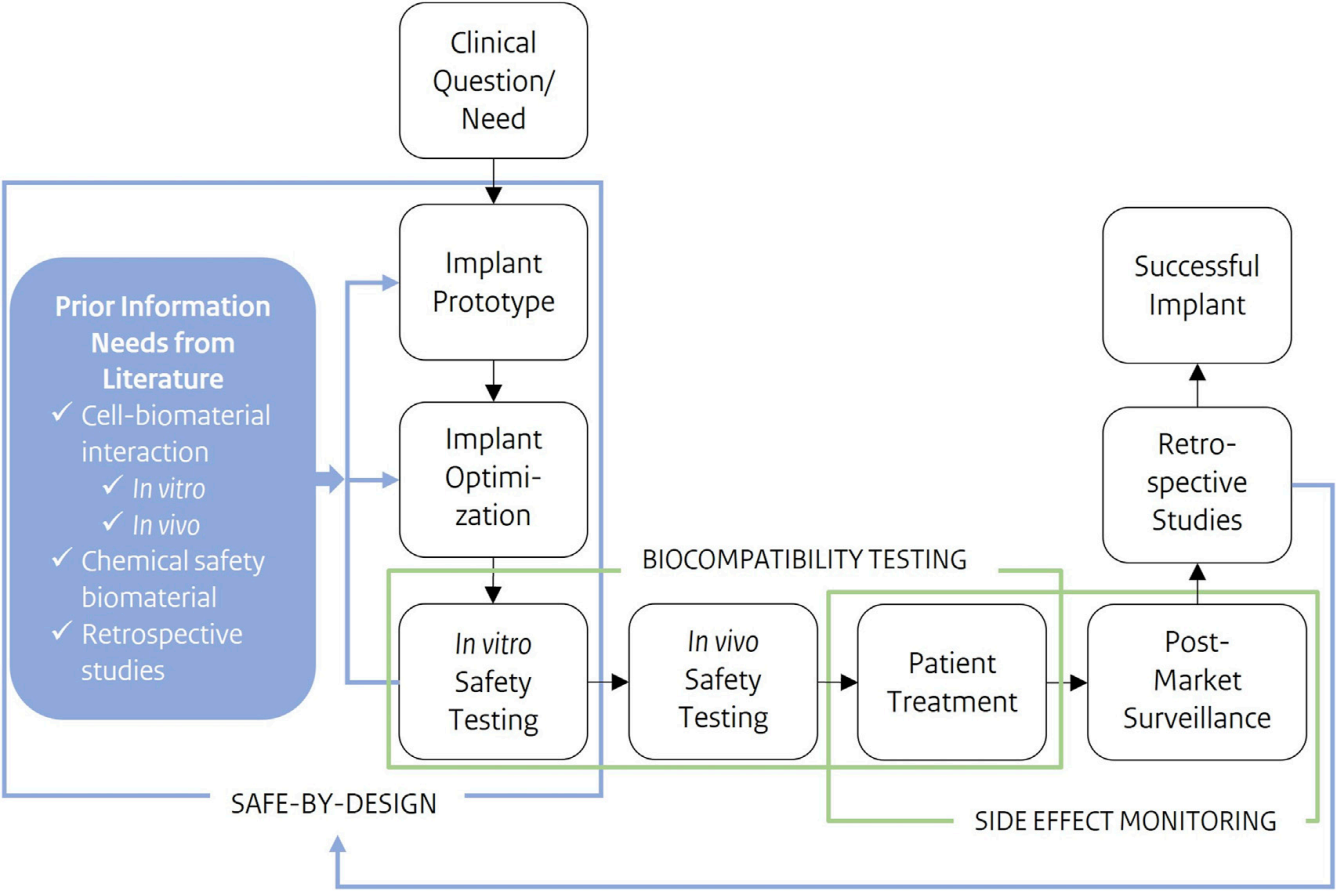
Schematic representation of a development process of a novel medical implant, from clinical questions/need as input to a successful implant as output. Safe-by-Design is applied early in the development process. Prior information needs, such as cell-biomaterial interactions or the chemical safety of components, are gathered preceding product development and optimization. These safety considerations are further integrated into implant development and optimization. Where safety testing results, feed design choices in implant prototype and optimization. Cell-biomaterial interactions are the prior information needs this study focusses on.

To bring the SbD approach for medical implants into practice, safety and functionality need to be integrated early into the development phase. Key safety aspects for the application specific implant need to be identified. The novel SbD strategy (Figure 1) for medical implants starts with a clinical need or question as input and a successful implant as a result. The very first SbD step is gathering implant and application specific information through existing literature on cell-biomaterial interactions, biomaterial chemical safety and retrospective studies. Additional information will be gathered through application specific *in vitro* assays. SbD aims to put safety parallel to implant optimization. By simultaneously characterizing the implant biomaterial and to test them for application-specific functionalities together with their biological safety, early in the process. Through this approach, complications later in the development process, e.g., during later stage *in vivo* tests, and ultimately in patients, might be avoided.

## 3 Cell-biomaterial interactions as safety aspect in design choices

### 3.1 Current regulatory framework and relevant standards

Before market entry, the biocompatibility of an implant, i.e., ‘the ability of a material to perform with an appropriate host response in a specific application’ (Williams, 2021), needs to be thoroughly investigated to minimize the risk of adverse effects. Requirements for the market entry of medical devices have been captured in the EU in the Medical Device Regulations (MDR), which aims to identify and monitor significant adverse events involving medical devices. The safety assessment and testing methods of (innovative) medical technologies are addressed in the standards of the International Organization for Standardization (ISO). The non-biological functionalities, (e.g., mechanical loading of a hip implant) and general biological safety are assessed. The general biological safety testing for medical implants is described in the ISO 10993 series. ISO 10993 addresses traditional toxicity endpoints, including separate standards for irritation, sensitization, cyto- and genotoxicity (ISO 10993, 2018). However, these standards mainly focus on the chemical safety of leachables of a biomaterial rather than direct biomaterial contact as occurs in the body, both for *in vitro* and *in vivo* testing. The exception is ISO10993-6, which is the standard for *in vivo* local effects after implantation, assessing tissue effects and interactions for the first time. Testing primarily leachables beforehand provides a standardized reflection of the chemical safety of the material and the manufacturing process. However, by solely relying on the leachables test *in vitro*, insights into the impact of implant material properties itself on the designated biological environment *in vitro* are missed and only will come across with *in vivo* testing of the whole product (Jurczak et al., 2024).

In addition to current practices, using existing knowledge on cell-biomaterial interactions as well as incorporating obtained *in vitro* cell-biomaterial interaction into safety assessment might provide insights predictive for the implant performance *in vivo* (Salthouse et al., 2022). This will lead to more targeted and probably fewer animal tests, as well as an increase in the overall success of the implant throughout the rest of the development process. ISO 10993 warrants characterization of the physical form and characteristics (e.g., geometry and surface roughness) of the material, however this is not yet related to any biological effect *in vitro* in the early stage testing. In the Safe-by-Design approach introduced here, existing *in vitro/vivo* information from literature and *in vitro* cell-biomaterial interaction assessment will be integrated into the early stages of development, allowing for directions that provide predictive insights into the implant’s *in vivo* performance.

### 3.2 Importance of *in vitro* cell-biomaterial interactions at early biomaterial development stages

The implant and the surrounding tissue form a system, where the interactions within the system are dependent on the implant biomaterial physical and chemical properties as well as the biological features of the surrounding tissue and host (Williams, 2019). The host response to an implant involves complex interactions between different cell types and properties of the biomaterial (Zhu et al., 2021; Rahmati et al., 2020). These cell-biomaterial interactions and subsequent signaling pathways are key in determining the tissue-specific compatibility of a biomaterial (Rahmati et al., 2020). For the (re)design of safe and functional implants, it is therefore essential to know the key biological processes involved in specific applications and the influence of biomaterial properties on these key processes on tissue and cellular level (Stich et al., 2022). It would be very valuable to take this knowledge into account in the safety standards, however, this state-of-the-art knowledge still needs to find its way to the existing and regulatory standards (Lackington et al., 2022).

Furthermore, different applications require different implant-tissue interactions, as these differ between, e.g., an orthopedic implant and a pacemaker. One needs to understand which key biological processes are relevant to consider, as well as their relationship with implant properties. Understanding this will give innovators directions towards safer and functional implants. Therefore, safety assessment should be based on the unique properties of the biomaterial and the intended use of the implant, where a SbD approach can help to guide innovators towards the most optimal biological outcome.

## 4 Biomaterial properties influencing the key biological processes for an orthopedic hip implant

To be safe and functional, an innovative antimicrobial orthopedic hip implant needs proper bone integration (osseointegration), an appropriate local immune response and minimize bacterial colonization on the implant surface. These three processes, i.e., osseointegration, immune response and bacterial adhesion, are here referred as the key biological processes. The processes are all connected to the prevalence of adverse outcomes related to hip implants, such as aseptic loosening or biomaterial-associated infection, making them valuable processes to investigate (Nobles et al., 2021).

Osseointegration is the stable anchorage of an orthopedic implant due to direct bone-to-implant contact without the interposition of nonbone tissue and is essential for the clinical success of orthopedic hip implants (Albrektsson and Johansson, 2001; Kim et al., 2017; Morinaga et al., 2009). Implants with proper osseointegration allow osteoblasts to adhere, proliferate and create a favorable microenvironment by secreting specific matrix proteins (Saldaña et al., 2011). It is regarded as one of the most decisive factors for long-term success of an hip implant and determined by several factors, such as biomaterial roughness (Stoilov et al., 2022).

The immune response related to implantation of a hip implant plays, in addition to osseointegration, a pivotal role in determining the clinical outcome (Salthouse et al., 2022). Upon implantation, implant biomaterials are recognized as foreign, initiating a complex cascade of events called the foreign body response (FBR). While a diverse set of immune cells and factors are involved in the FBR (Christo et al., 2015) this review focusses on macrophages since they are important players in the initial phase of inflammation (Batool et al., 2021). Macrophages adapt to their local microenvironment, influenced by biomaterial properties, such as wettability and roughness, and can then polarize to different phenotypes (Chen et al., 2022). Tissue damage due to implantation leads to an initial inflammatory response with more pro-inflammatory phenotyped macrophages. On the contrary, a more anti-inflammatory macrophage phenotype promotes healing and regeneration of the tissue (Xie et al., 2020). Of importance is a timely switch from pro-towards anti-inflammatory to allow for the formation of new bone tissue (He et al., 2020). As such this first macrophage response is a determinant for the biological outcome (Salthouse et al., 2022). Implant biomaterial design should thus take into account the macrophage response, and optimize this by tweaking the biomaterial properties (Bu et al., 2022; Dong et al., 2022).

Biomaterial-associated infections remain a major challenge in designing and developing orthopedic hip implants (Pandey, 2022). Bacterial adhesion is a complex process where different type of physical-chemical interactions are involved, these interactions are dependent on bacterial and biomaterial properties (Kreve and Reis, 2021; Filipović et al., 2020; Yang et al., 2022). The process begins with a reversible and unstable adhesion phase, followed by the second phase where bacterial firmly anchor to the surface and form a biofilm (Dong et al., 2022; Filipović et al., 2020; Yang et al., 2022). In biofilms, bacteria are embedded in a protective matrix, consisting of extracellular polysaccharides, matrix proteins and extracellular DNA, where they can survive even under harsh conditions (Li et al., 2023). Bacteria in biofilms are difficult to treat with conventional antibiotics due to limited penetration into the protective biofilm matrix, metabolically reduced phenotypes and the development of antibiotic resistance. Implant surface design with antimicrobial functionalities which minimize bacterial adhesion, such as non-adhesive or bactericidal surfaces, can aid in the prevention of biomaterial-associated infections. Antimicrobial implant technologies differ in biomaterial properties that have an influence on key biological processes, thus influence the safety as well as the functionality of the orthopedic hip implant (Yang et al., 2022).

**FIGURE 2:**
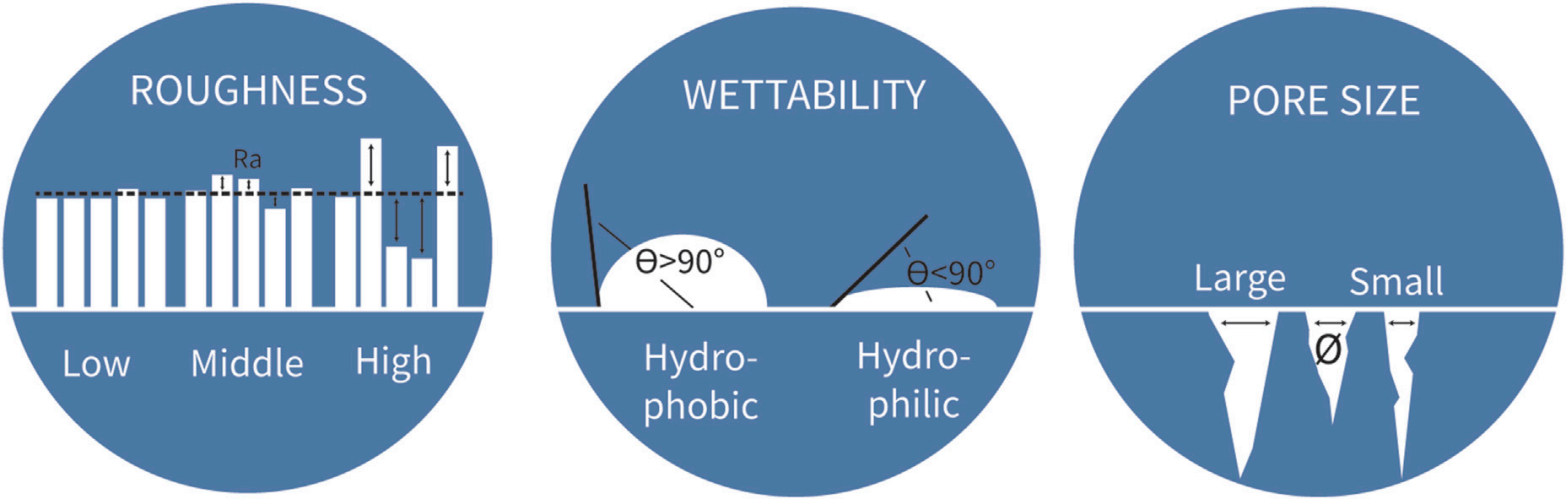
Three biomaterial properties to consider for implant biomaterial design. Measure for roughness is the average peaks and valleys expressed (Ra) in the unit µm. The angle of a water drop determines the wettability of the biomaterial. The unit for pore size is the average diameter of the pores.

These three key biological processes, osseointegration, immune response and bacterial adhesion, collectively contribute to the safety and functionality of the hip implant. Therefore, all processes should be considered simultaneously during implant design and optimization to minimize adverse outcomes. The biological response to an implant is predominantly influenced by its biomaterial surface properties (Chen et al., 2016). For instance, the physicochemical properties of the biomaterial surface directly affect the cellular behavior of the surrounding tissue (Ren et al., 2021). Important physicochemical surface properties of titanium hip implant specifically are roughness, wettability and pore size (Figure 2) (Ren et al., 2021). The relevance of each of these surface properties is addressed in more detail below.

### 4.1 Influence of roughness on the selected key biological processes

**FIGURE 3:**
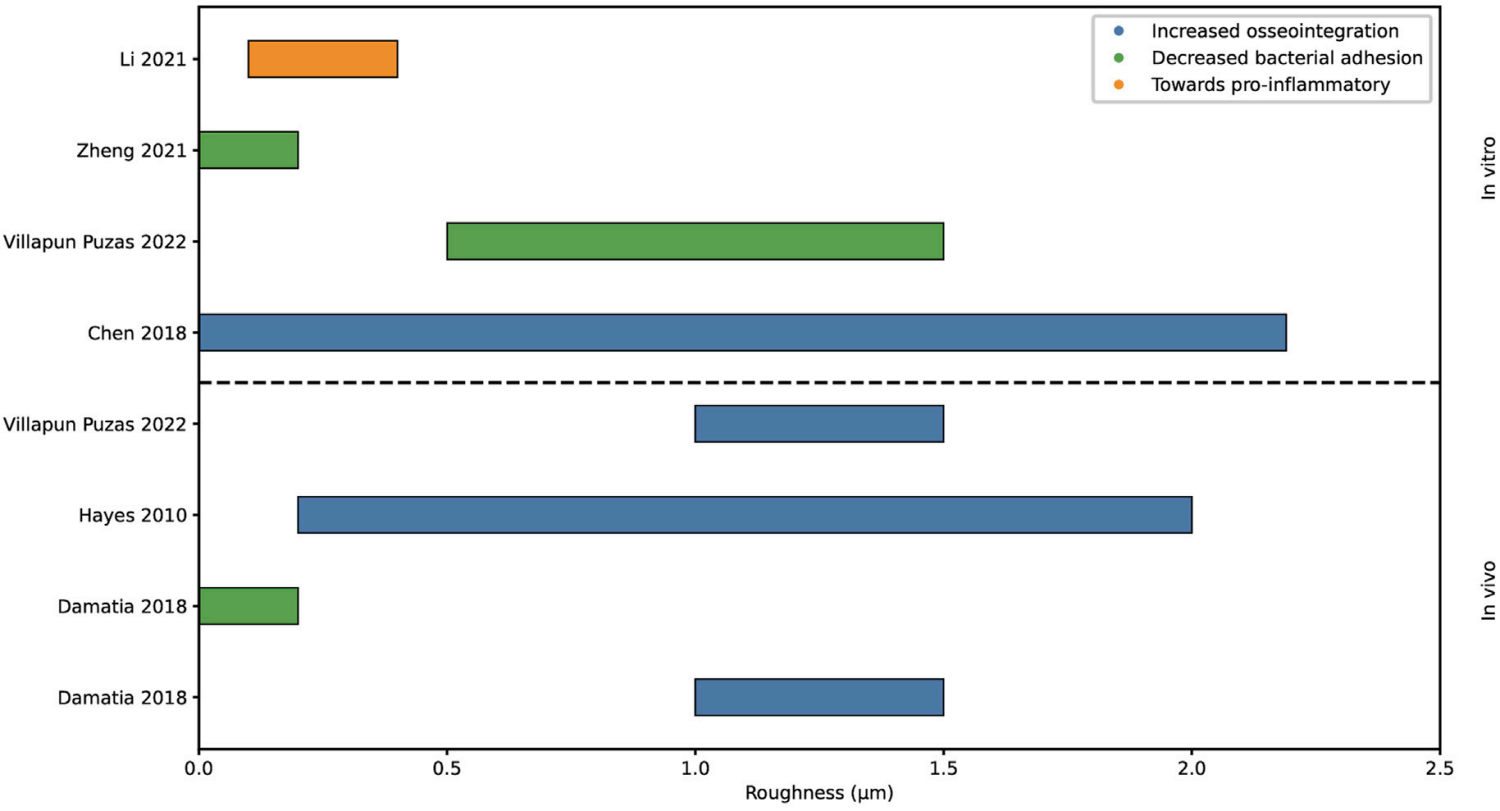
Graphical representation of the proposed surface roughness values for increased osseointegration, decreased bacterial adhesion and polarization macrophage. Analyzed with *in vivo* and *in vitro* methods.

Roughness is a measure of the texture of a surface, expressed in average peaks and valleys as the profile roughness parameter Ra, with µm as unit (Figure 2). This surface property has been researched extensively in relation to osseointegration and bacterial adhesion. In literature, roughness is distinguished at the micro and nanoscale. The microscale roughness dictates tissue level interactions and improves mechanical anchorage of the implant in the bone tissue. Nanoscale roughness activates biological responses at the cell and protein level (Nobles et al., 2021; Stich et al., 2022; Albrektsson and Wennerberg, 2019). Figure 3 summarizes the findings of eight literature reviews, where the upper part of the graph shows *in vitro* data and the lower part *in vivo* data.

Hayes et al. hypothesized about a so-called roughness window. Within this effective roughness window, which ranges from 200 to 2000 nm, cells reacted optimally resulting in increased osseointegration (Hayes and Richards, 2010). Chen et al. spoke of an optimum range of roughness as well, showing that a roughness over 2.19 µm inhibits osteoblastic adhesion. Therefore, a roughness below 2.19 µm is considered to stimulate osseointegration (Chen et al., 2018; Anselme et al., 2000).

On the other hand, increased roughness increases the colonization of bacteria (Yang et al., 2022). The grooves can provide shelter for bacteria against antibiotic treatment (Damiati et al., 2018). Villapun Puzas et al. also stated that bacterial attachment generally increased with an increasing roughness, additionally they specified that a roughness between 0.5 and 1.5 µm achieves a limited colonization of bacteria (Villapun et al., 2022). Whereas Zheng et al and Damiati et al. observed limited bacterial adhesion at a roughness below 0.2 µm, however Zheng et al states that a so called threshold roughness of 0.2 µm is currently debatable due to contradicting studies (Filipović et al., 2020; Damiati et al., 2018; Zheng et al., 2021).

Li et al. explored the findings for the influence of roughness onto the immune response and found *in vitro* data suggesting a macrophage phenotype polarization towards pro-inflammatory with increasing roughness from 100 to 400 nm (Li et al., 2021).

Altogether, several studies have proposed an optimal roughness window for osseointegration, demonstrating an consensus of optimal roughness values within literature as can be seen in Figure 3. However, there is no agreement between studies on the optimal roughness value for reduced bacterial adhesion. *In vitro* research has explored the macrophage response to roughness, yet the identification of an optimal roughness value for an optimal immune response remains elusive.

### 4.2 Influence of wettability on the selected key biological processes

**FIGURE 4:**
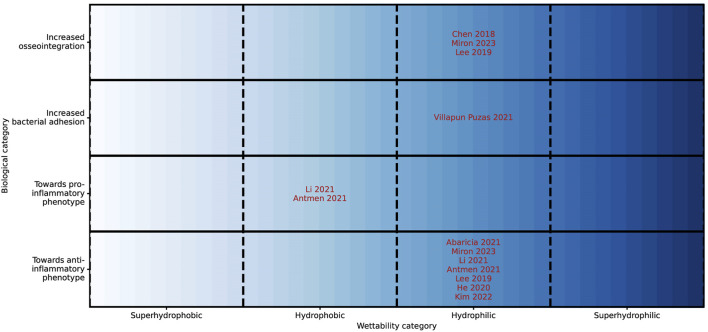
Graphical representation of influence of wettability categories onto the biological categories osseointegration, bacterial adhesion and polarization of macrophages. The location of the author represents the combination of the categories placed on the y- and x-axis. For example, Villapun Puzas et al. described an increase in bacterial adhesion for hydrophilic surfaces. All studies represent *in vitro* methods.

Wettability is defined as the ability of a material to maintain contact with a liquid. It is expressed as the contact angle of a drop of water; a hydrophilic surface exhibits an angle below 90°, whereas hydrophobic surfaces have a water contact angle greater than 90° (Figure 2) (Nobles et al., 2021; Damiati et al., 2018). The wettability of a surface is a major driving force for the adsorption of proteins (Barberi and Spriano, 2021; Mariani et al., 2019) which plays a critical role in mediating tissue integration outcomes for implants, influencing cell and bacteria adhesion and proliferation on the implant biomaterial surface immediately post-implantation (Barberi and Spriano, 2021). The spatial conformation of proteins is very different between hydrophobic and hydrophilic surfaces due to protein unfolding, resulting in different biochemical and physicochemical behavior (Barberi and Spriano, 2021; Mitra, 2020). All included reviews covered *in vitro* studies only (Figure 4).

All three studies on osseointegration align and provide evidence that hydrophilic surfaces promote osseointegration (Chen et al., 2018; Miron et al., 2023; Lee et al., 2019).

Villapun Puzas et al. have found however, that also bacteria have an increased proliferation on hydrophilic surfaces compared to hydrophobic surfaces (Villapun et al., 2022). Several studies have explored wettability as a strategy to reduce bacterial attachment. Zheng et al. presented that superhydrophobic and superhydrophilic surfaces serve as effective means against bacterial adhesion (Zheng et al., 2021), which are not displayed in Figure 4. They noted that findings on the influence of wettability on bacterial adhesion are inconsistent. Moreover, this relationship is influenced by the hydrophobicity of bacteria, which differs among species. Generally, hydrophobic bacterial tend to adhere to hydrophobic surfaces, while hydrophilic bacteria prefer hydrophilic surfaces (Filipović et al., 2020).

Wettability and the related protein adsorption are a big driving force for macrophage polarization. Several literature reviews describe that hydrophilic surfaces drive the macrophage polarization towards an anti-inflammatory phenotype *in vitro* (He et al., 2020; Li et al., 2021; Miron et al., 2023; Lee et al., 2019; Abaricia et al., 2021; Antmen et al., 2021; Abaricia et al., 2020). Moreover, Li et al. and Antmen et al. presented literature that found an increase in pro-inflammatory phenotype factors as a reaction to hydrophobic surfaces (Li et al., 2021; Antmen et al., 2021).

In conclusion, hydrophilic surfaces promote cellular and bacterial adhesion through the appropriate spatial conformation of proteins, and stimulate an anti-inflammatory environment for macrophages.

### 4.3 Influence of pore size on the selected key biological processes

Porous materials or porous surface coatings help promote cellular attachment, vascularization and transport of nutrients and thus porosity plays an essential role during the early stages of osseointegration (Bandyopadhyay et al., 2023). Porosity increases the surface area for potential cell adhesion and helps with the interlocking between host tissue and implant. Porosity is defined as a measure of spaces in a material, where the pore size is the average diameter of the spaces (Figure 2). The percentage of porosity and the size of the pores have limitations. During implant design it should be considered that pores and porosity throughout the structure decrease structural stability. Therefore, porous structures should be tested on their mechanical strength to withstand *in vivo* stresses (Zhu et al., 2021; Bandyopadhyay et al., 2023; Falchete do Prado et al., 2018). The size of the pores of a biomaterial dictate the cellular interactions; small pore sizes limit cellular migration into the material, therefore cells tend to grow only on the outer surfaces. Larger pore sizes restrict the total surface area for cells to attach to but stimulate cellular migration into the biomaterial (Chen et al., 2018). Thus, this would suggest there is a pore size range where cellular migration, adhesion and vascularization is optimal.

Chen et al. and Nobles et al. stated that an appropriate pore size is between 100–600 μm and 100–700 µm respectively, for cell ingrowth and adhesion (Nobles et al., 2021; Chen et al., 2018). Chen et al. also noted favorable pore sizes of around 100–135 µm for osteoblast adhesion. However, it's important to clarify that the optimal pore size for bone regeneration may not necessarily align with that for osteoblast adhesion (Chen et al., 2018). Whereas Gu et al. reviewed several studies of porous structures in animal models (Gu et al., 2022). Their findings suggested pore sizes ranging from 500 to 600 µm and a porosity of 80%–90% is most optimal for osseointegration on titanium scaffolds tested *in vivo*. Karageorgiou et al. observed increased osteogenesis for scaffolds with pore sizes above 300 μm, due to the possibility of vascularization (Karageorgiou and Kaplan, 2005). They pointed out that the upper limit of scaffold pore sizes and porosity is set by mechanical restraints. Hussain et al. found macroporous scaffolds, with a pore size range from 250 to 500 μm, to be essential for bone regeneration due to the proper osteoblast attachment, angiogenesis and integration of host tissue (Hussain et al., 2024). While Hussain et al.’s findings are primarily oriented towards tissue engineering applications rather than orthopedic hip implants, there exists potential to learn from this field.

The porosity of biomaterials can be designed to influence the macrophage phenotype, Lee et al. found an increase in anti-inflammatory macrophages with pores of diameters between 100 and 200 nm (Lee et al., 2019). He et al. stated that pores and porosity influence oxygen supply after implantation by affecting vascularization. Limited oxygen supply results in inflammation affecting the bone remodeling. They found an anti-inflammatory phenotype with increasing pore size (He et al., 2020). However, as Nobles et al. raised, the porosity and pore size should be optimized for its intended application, dependent on important biological processes, since there is no “one-size-fits-all” (Nobles et al., 2021).

**FIGURE 5:**
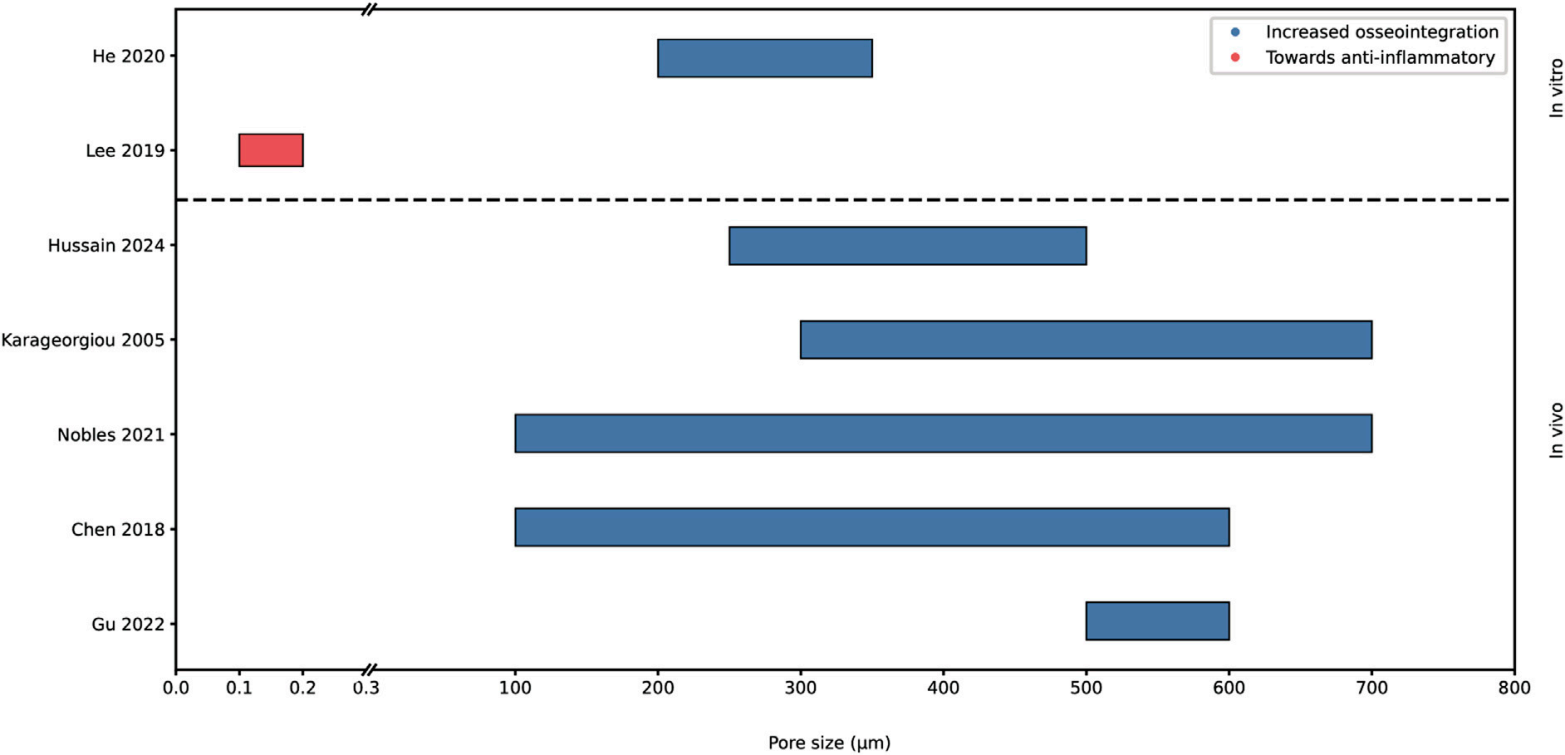
Graphical representation of proposed optimal pore sizes for increased osseointegration and observed polarization of macrophage. Analyzed with *in vivo* and *in vitro* data. The x-axis is broken up into 0-0.3 µm and 0.3-800 µm.

Figure 5 shows both *in vitro* and *in vivo* research investigating the impact of pore sizes onto the key biological processes. In conclusion, the impact on osseointegration has been extensively studied, revealing overlapping findings. However, the research into the effect on bacterial adhesion and macrophage polarization has been limited.

## 5 Safe-by-design for novel antimicrobial hip implants

The relationship between biomaterial properties and the key biological processes is crucial for determining the clinical success of an implant. A better understanding of the cell-biomaterial interactions can greatly contribute to the implant design phase. Therefore, this review has examined existing literature reviews to identify possible safe innovation ranges. Extensive research has been done on the effect of biomaterial properties on osseointegration, finding consistent ranges of optimal biomaterial properties. Contrarily, no consistent information could be found regarding bacterial adhesion and immune response, except for hydrophilic surfaces promoting anti-inflammatory phenotypes in macrophages. In the Safe-by-Design approach, the gathered information helps to identify potential hazards or innovation windows early on (Dekkers et al., 2020), as summarized in Table 1. The cells of Table 1 are color categorized on the level of evidence found supporting the identified interactions displayed in the table cells.

**TABLE 1:** Overview on relation between the biomaterial property design space as related to the three key biological processes. In context of Safe-by-Design the results are categorized in convincing (green), indication (orange) and inconclusive (red) information.

Surface property	Increased osseointegration	Decreased bacterial adhesion	Immune response
Roughness (Ra)	1.0–1.5 µm	<0.2 µm	Inconclusive
Wettability	Hydrophilic	Inconclusive	Hydrophilic = towards anti-inflammatory phenotype
Pore size (D)	300–500 µm	Inconclusive	Inconclusive

This literature review reveals that while individual biomaterial property optima may be present for distinct biological processes, there is insufficient data to conclude on an optimal range to satisfy all key biological processes. Yet, these insights provide directions for safer and functional biomaterial innovations and form the first step in a Safe-by-Design framework. As a next step, the impact of the biomaterial properties on key biological properties should be tested using various *in vitro* assays, to provide more detailed insight and reduce uncertainty (Salthouse et al., 2022). The SbD approach is a balancing act between different aspects. The approach helps to better understand potential negative impacts of design choices and provides innovators the option to discuss trade-offs between different material impacts.

## 6 Discussion: Safe-by-Design for medical implant innovation

For innovators to successfully apply a SbD strategy, information is key. The more knowledge available and gathered in an early stage of innovation, the more efficient the product development and safety assessment can be performed. Where retrospective studies, focused on existing implant material design (Puijk et al., 2023), can provide valuable insights by creating a feedback loop from existing data to inform and improve future implant innovations. As mentioned, there exists no optimal property value to maximize all biological processes. However, this knowledge can function as a guide during the design phase and safety assessment. As seen for orthopedic hip implants, if the biomaterial roughness deviates for the optimal osseointegration roughness range, aimed at limiting bacterial adhesion, safety assessments should prioritize evaluating the osseointegration capabilities of the biomaterial. Conversely, if the design is within the optimal osseointegration roughness range but also promoting bacterial adhesion, additional preventive (designing) strategies may be considered to fight bacterial adhesion. The SbD approach can be applied to any implant type or application, where different key biological processes are involved and the properties to be varied are dependent on the type of biomaterial used.

In this manuscript, we highlighted a selection of three material properties within the design space and three biological processes that are acutely influenced by these properties. However, long-term effects, such as biomaterial degradation, must also be considered, as they can significantly impact tissue reactions. For instance, while metal wear particles from metal-on-metal hip implants can cause adverse effects (Dapunt et al., 2014), bioactive degradation products may actively support tissue regeneration (Rahaman et al., 2014). This interplay between material properties and biological responses illustrates the complexity of biomaterials and medical implants. Although the current study focused on a Safe-by-Design (SbD) approach for the surface properties of titanium orthopedic implants, the challenges are likely to grow with the development of more innovative biomaterials and tissue-engineering constructs. These may involve combinations of materials with distinct properties, the integration of bioactive components, or even cell-loaded constructs (Todros et al., 2021).

**FIGURE 6:**
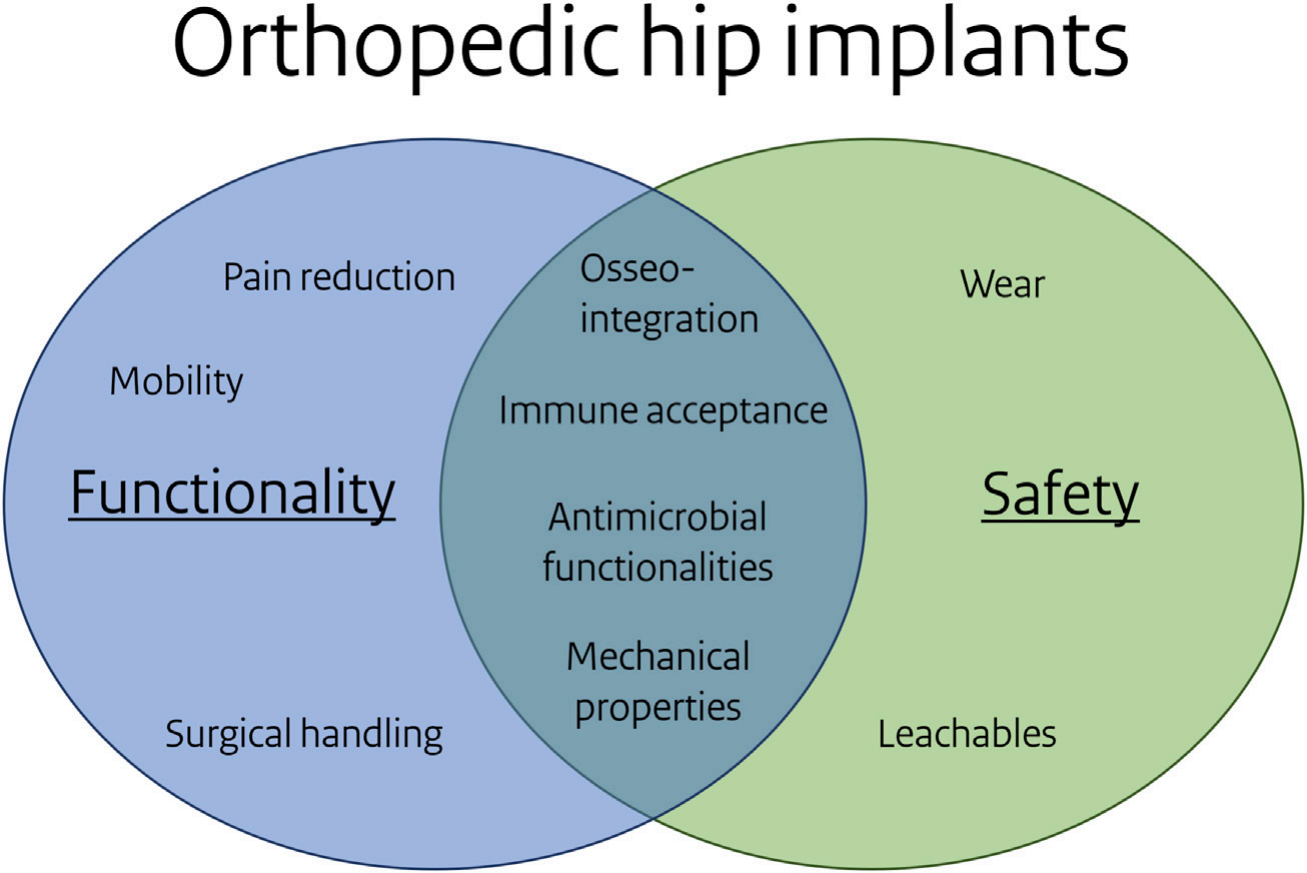
Overlap of functionality and safety for the orthopedic hip focus application. Note: some items under functionality and safety can be related, e.g. particular leachables reduce bacterial adhesion or affect osseointegration, mechanical properties may link to wear.

In nanomaterial research, Tavernaro et al. (Tavernaro et al., 2021) stated “A Safe-by-Design strategy strives for negligible human safety risks through an acceptable balance between safety, product functionality and, as far as possible, costs”. The implementation of SbD in various industries often involves substituting harmful compounds with less harmful alternatives, sometimes at the expense of product functionality. In contrast, implants present a case where safety and functionality are inherently interconnected. Implant functionalities, such as an antimicrobial surface, contribute to implant safety by avoiding biomaterial-associated infections (see Figure 6). For this focus-application, the common causes of implant failure are implant loosening and infection (Khalifa and Bakr, 2021). These causes are linked to the selected key biological processes and are relevant to both safety and functionality. Paying attention to the influence of biomaterial properties on these key biological processes during the design and development process, within a SbD approach, will increase the safety of implants. Figure 6 illustrates the phenomena associated with functionality, safety, or both. Phenomena like osseointegration, immune acceptance and antimicrobial functionalities contribute to both functionality and safety.

This review is not systematic, but illustrates the use of the SbD framework and its potential outcomes. Search strings were used to identify material property–biological response relationships, with exclusions like mandibular implantation, skull implantation, and induced animal models. While more data is needed for firm conclusions, the findings offer a starting point for biomaterial innovation using Safe-by-Design. The methodology relies predominantly on literature reviews and articles that analyzed data and identified trends within a specific property range. Consensus could be found for optimal property ranges for osseointegration in literature reviews. However, the influence of the selected properties on macrophage polarization and bacterial adhesion lacked sufficient clarity to do so. Only a few reviews identified specific property ranges that reliably induced a particular biological outcome for these two processes, resulting in limited inclusion of information in this review. This highlights the uncertainty surrounding the relationship between biomaterial properties and macrophage polarization and bacterial adhesion, emphasizing the necessity for further research in this area. Additionally, variability exists among the studies included in the literature reviews discussing in this manuscript. This variability includes the type of tests used, *in vitro* or *in vivo* methods, and the choice of animal model or cell types. The studies focused on different biomaterials, primarily titanium, with an emphasis on design considerations for specific applications. Since not all properties can be modified on titanium alone, the scope includes coatings and similar solutions. As a result, mapping the influence of biomaterial properties on several biological processes for one particular application is challenging. Nevertheless, these overviews of existing data can give an indication on the influence of one biomaterial property on several important biological processes. The raised variability observed between studies also highlights the need for standardizing the gathering and reporting of findings. The use of different models, measurement tools and techniques can greatly impact the results. Further information on the included studies can be found in the Supplementary Table S1.

A remaining challenge for *in vitro* safety assessment, is the lack of optimized and validated methods for the complex evaluation of key biological processes (Przekora, 2019). The standardized *in vitro* and *in vivo* tests described in ISO 10993 are usually one of the last steps in the process before patient trials. The *in vitro* methods mostly concern leachables and therefore cannot address the complexity of the direct interaction between the biomaterial and the host (Williams, 2017). To better understand the local events at the implant biomaterial surfaces, more advanced and application-specific *in vitro* tests are necessary, e.g., a standardized *in vitro* test for osseointegration assessment (Antmen et al., 2022). These can be used in the SbD approach after consideration of the existing information related to biomaterial properties and key biological processes. Furthermore, understanding biomaterial properties and the associated host tissue responses will guide the choice of biocompatibility tests. *In vitro* testing does not replace *in vivo* testing in animals and in humans for medical device approval. However, this can function as a preliminary evaluation to identify and address safety risks and hazard alerts during the development process, minimizing the number and expense of *in vivo* testing (Bernard et al., 2018).

## 7 Conclusion

During implant design, numerous biomaterial properties can be fine-tuned to align with the key biological processes pivotal for the success of the implant within a specific application. A comprehensive understanding of this intricate relationship between biological processes and biomaterial properties is crucial to optimize both components synergistically. A Safe-by-Design approach supports innovators during the design phase, providing essential tools to develop implants that prioritize safety at the beginning of development. This study introduced the application of Safe-by-Design for orthopedic titanium hip implants, emphasizing the advantages from early-stage knowledge integration. One important aspect is the relationship between biomaterial properties (roughness, wettability and pore size) and key biological processes (osseointegration, immune response and bacterial adhesion), serving as a cornerstone in implant design that yield optimal outcomes. From literature, this study has demonstrated that for certain combinations of biomaterial properties and key biological processes, an optimal property value for an optimal biological response can be found. Such a range was hypothesized by various authors for roughness and pore size, influencing osseointegration. Hydrophilic surfaces seem to drive an anti-inflammatory phenotype, combined with increased osseointegration and unfortunately increased bacterial adhesion. Consistent information regarding the impact of pore size and surface roughness on bacterial adhesion and macrophage polarization could not be found. A property range to satisfy all key biological processes has not been identified. This complex interaction between biomaterial properties and biological processes emphasizes the need for a careful Safe-by-Design approach in the precise design of medical implants. In Safe-by-Design the prior information needs focus on application-specific key biological processes linked to common adverse outcomes, as well as those crucial for implant success and safety. Application-specific *in vitro* tests will be selected and applied aiming to optimize the implant biomaterial and select the most suitable candidate for future *in vivo* studies, saving time and resources. Collectively, these steps can serve to guide design and innovation.
