# Supplementary material for: Utilizing biomaterial surface properties to improve orthopedic hip implant safety and function in a Safe-by-Design approach
Source: Front Bioeng Biotechnol. 2025 Feb 21;13:1504883. doi: 10.3389/fbioe.2025.1504883 (PMC11885263; doi:10.3389/fbioe.2025.1504883)
Supplement: Supplementary file 1 [file DataSheet1.pdf]

Supplementary Material

1 Supplementary Figures and Tables

Table 1 List of data included in the roughness, wettability and pore size graphs. Literature search carried out 01/2024 – 05/2024.

Author	Material property	Property value (μm)	Material type	Biological process	Based on	Model (animal / cell type / bacterial species)
Chen 2018 **(39)**	Pore size	100-600	Titanium	Cell ingrowth and adhesion	Review	-
Nobles 2021 **(5)**	Pore size	100-700	Titanium	Bone tissue-ingrowth	In vivo	Male Sprague-Dawley rats; Hybrid male dogs;
Karageorgiou 2005 **(55)**	Pore size	>300	Titanium; Hydroxyapatite	Better osteogenesis	In vivo	Rabbit transcortical model;
Gu 2022 **(54)**	Pore size	500-600	Titanium	Osteogenesis	In vivo	Several animal models
Hussain 2024 **(56)**	Pore size	250-500	Hydroxyapatite	Osseointegration	In vivo	Mature wethers (goat)
Lee 2019 **(48)**	Pore size	0.1-0.2	Anodic alumina	Towards anti-inflammatory	In vitro	-
He 2020 **(30)**	Pore size	200-350	Scaffold collagen-glycosaminoglycan	Optimal for bone tissue engineering	In vitro	MC3T3-E1
Damiati 2018 **(40)**	Roughness	1-1.5	Titanium	Implant fixation	Review	-
Damiati 2018 **(40)**	Roughness	<0.2	Titanium	Negative bacterial adhesion	Review	-
Hayes 2010 **(38)**	Roughness	0.2-2	Titanium	Osseointegration	In vivo	-
Chen 2018 **(39)**	Roughness	>2.19 inhibits osteoblastic cell adhesion	Titanium	Osseointegration	In vitro	Isolated human bone cells explants

Villapun Puzas 2022 **(41)**	Roughness	0.5-1.5	Titanium	Limited colonization	In vitro	S. mutans.
Villapun Puzas 2022 **(41)**	Roughness	1-1.5	Titanium	Cell attachment for mammalian cells	Review	-
Zheng 2021 **(42)**	Roughness	<0.2	Composite resin surfaces	Negative bacterial adhesion	In vitro	S. mutans; Streptococcus sobrinus;
Li 2021 **(43)**	Roughness	Increasing roughness	Titanium	Towards pro-inflammatory	In vitro	RAW 264.7 macrophages
Chen 2018 **(39)**	Wettability	Hydrophillic	Hydrophilic coatings	Cellular adhesion; osseointegration	In vitro	MC3T3-E1; Saos-2;
Villapun Puzas 2022 **(41)**	Wettability	Hydrophillic	Titanium	Proliferation of bacteria and mammalian cells	In vitro	MG-63;
Abaricia 2021 **(49)**	Wettability	Hydrophillic	Titanium	Modulate anti-inflammatory macrophage activation	In vitro	-
Miron 2023 **(47)**	Wettability	Hydrophillic	Titanium	Towards anti-inflammatory	In vitro	Rodent bone marrow-derived macrophages
Li 2021 **(43)**	Wettability	Hydropobic	Untreated polystyrene	Towards pro-inflammatory	In vitro	Peritoneal macrophages
Li 2021 **(43)**	Wettability	Hydrophillic	Titanium	Towards anti-inflammatory	In vitro	Primary murine macrophages
Antmen 2021 **(50)**	Wettability	Hydrophobic	Titanium	Towards pro-inflammatory	In vitro	Primary murine macrophages
Antmen 2021 **(50)**	Wettability	Hydrophillic	Titanium	Towards anti-inflammatory	In vitro	Primary murine macrophages
Lee 2019 **(48)**	Wettability	Hydrophillic	Titanium	Osseointegration	In vitro	Primary human osteoblasts
Lee 2019 **(48)**	Wettability	Hydrophillic	Titanium	Towards anti-inflammatory	In vitro	RAW 264.7 macrophages
Kim 2022 **(62)**	Wettability	Hydrophillic	Titanium	Towards anti-inflammatory	In vitro	RAW 264.7 macrophages; Rodent bone marrow-derived macrophages;

He 2020 **(30)**	Wettability	Hydrophillic	Titanium	Towards anti-inflammatory	In vitro	Primary murine macrophages; RAW 264.7 macrophages
